# Is RAS the Link Between COVID-19 and Increased Stress in Head and Neck Cancer Patients?

**DOI:** 10.3389/fcell.2021.714999

**Published:** 2021-07-15

**Authors:** Anem Iftikhar, Mohammad Islam, Simon Shepherd, Sarah Jones, Ian Ellis

**Affiliations:** Unit of Cell and Molecular Biology, The Dental School, University of Dundee, Dundee, United Kingdom

**Keywords:** stress, COVID-19, cancer, oral cancer, ACE2, renin angiotensin system, head and neak cancer

## Abstract

The COVID-19 pandemic emerged as a largely unexplained outbreak of pneumonia cases, in Wuhan City, China and rapidly spread across the world. By 11th March 2020, WHO declared it as a global pandemic. The resulting restrictions, to contain its spread, demanded a momentous change in the lifestyle of the general population as well as cancer patients. This augmented negative effects on the mental health of patients with head and neck cancer (HNC), who already battle with the stress of cancer diagnosis and treatment. The causative agent of COVID-19, SARS-CoV2, gains entry through the Angiotensin converting enzyme 2 (ACE2) receptor, which is a component of the Renin Angiotensin System (RAS). RAS has been shown to influence cancer and stress such that it can have progressive and suppressive effects on both. This review provides an overview of SARS-CoV2, looks at how the RAS provides a mechanistic link between stress, cancer and COVID-19 and the probable activation of the RAS axis that increase stress (anxiogenic) and tumor progression (tumorigenic), when ACE2 is hijacked by SARS-CoV2. The mental health crises brought about by this pandemic have been highlighted in many studies. The emerging links between cancer and stress make it more important than ever before to assess the stress burden of cancer patients and expand the strategies for its management.

## Introduction

COVID-19 emerged as a largely unexpected and unexplained outbreak of pneumonia cases, in Wuhan City, China and rapidly evolved into an epidemic. In January 2020, WHO named the causative virus as Severe Acute Respiratory Syndrome Corona Virus 2 (SARS-CoV2), previously known as 2019 novel coronavirus (2019-nCOV) and declared the epidemic as a public health emergency of international concern. In February 2020, this outbreak was named as Coronavirus disease 2019 (COVID-19) (Sun et al., 2020; Wang M.Y. et al., 2020). By 11th March 2020 the World Health Organization declared a global pandemic. As of 1st May 2021, there are 150,989,419 confirmed cases and 3,173,576 confirmed deaths globally reported to WHO.

The emergence of COVID-19 pandemic presented an unprecedented global health challenge. Its ability to spread rapidly necessitated tough lockdown restrictions, which spurred uncertainty in all segments of society. Social isolation, physical distancing from loved ones, closure of places of entertainment, job losses, fear of infection, uncertainty about COVID-19 treatment effectiveness are all causes of anxiety in the general population. Mental health and well-being have been and probably will be, adversely affected across all age groups (Galea et al., 2020). A meta-analysis including studies up to May 2020, indicated that 24.4% individuals from the general population suffered psychological distress (Cooke et al., 2020).

Head and Neck Cancer (HNC) patients already suffer emotionally due to the unique set of challenges they face. Significant functional, personal and cosmetic attributes are all affected by disfiguring surgery in the region. The diagnosis itself, debilitating course of the disease, complex treatment plans and functional disability act as psychological stressors that reduce the quality of life for patients with HNC (Iftikhar et al., 2021). Fear of recurrence also weighs heavily on the patient. The COVID-19 outbreak has introduced layers of stress on top of the potentially challenging psychological health of patients with HNC. Cancer has been found to be a contributory factor in 20% of COVID-19 deaths (Kuderer et al., 2020; Ng D.W.L. et al., 2020; Onder et al., 2020; Williamson et al., 2020; Xia et al., 2020; Fung and Babik, 2021). Early studies from the COVID-19 pandemic indicated that 1 in 3 cancer patients suffered from psychological distress, suggesting a higher number in comparison to pre-pandemic studies (Klaassen and Wallis, 2021). This review looks at how the COVID-19 pandemic has amplified stress in cancer patients and how stress, cancer and COVID-19 may interplay, mechanistically, via the Renin Angiotensin System (RAS).

**FIGURE 1:**
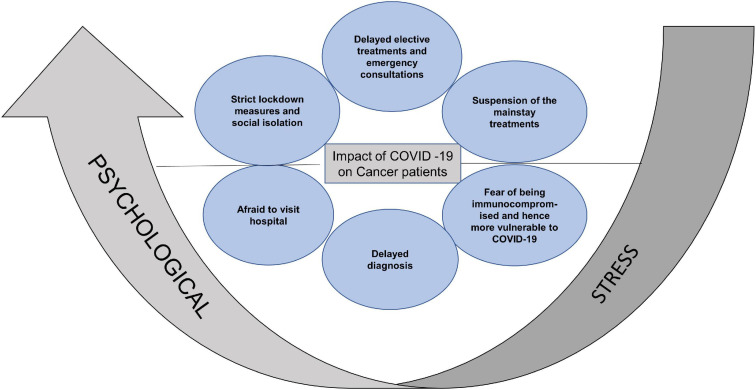
Summary of the reasons for aggravated psychological stress in cancer patients. Strict lockdown measures, social isolation, delayed consultations, suspension of mainstay treatments, fear of increased vulnerability to COVID-19 and limited access to hospital, increased the psychological stress in cancer patients.

In addition to the burdensome dynamics of a cancer diagnosis and treatment, there are risks of severe complications of COVID-19 infection secondary to compromised immunity due to malignancy or the anticancer treatments. The adaptation and re-organization of traditional health care delivery to minimize risk of SARS-CoV2 infection, has caused significant disruption to the delivery of cancer surgery, chemotherapy and radiation. Some patients were unable to continue their prescribed treatment due to the stringent lockdown measures and its socioeconomic consequences (Gelardi et al., 2020; Ratnasekera et al., 2020). Substitution of face-face consultations by video or phone were also perceived as a barrier to seeking medical advice. Late diagnosis and delays in treatment impact the survival in HNC patients and therefore delays due to the COVID-19 pandemic are anticipated to affect the disease outcome. All of this, along with the recommendation for more intensive shielding, in comparison to the general population, amplified the pre-existing psychological burden of cancer patients (Park et al., 2020; Figure 1).

Fear and anxiety in cancer patients during the pandemic has been widely explored (Ng K.Y.Y. et al., 2020; Swainston et al., 2020; Patni et al., 2021). Wang Y. et al. (2020) from China reported that out of 6213 cancer patients, 24.4% had depression, 17.7% had anxiety, 9.3% had post-traumatic stress disorder and 13.5% had hostility. In another study by Wuhan University, 86.5% reported fear of disease progression, 65% reported anxiety and 74.5% reported depression (Chen G. et al., 2020). Romito et al. (2020) from Italy, reported 36% patients of lymphoma with anxiety, 31% with depression and 36% with post-traumatic stress disorder (PTSD), as analyzed through Hospital Anxiety and Depression Scale (HADS-A) and (HADS-D). Increased anxiety, insomnia and depression in breast cancer patients was observed using the HADS, Insomnia Severity Index, Generalized Anxiety Disorder Questionnaire (GAD-7) and Impact of Events Scale Revised (IES-R) (Juanjuan et al., 2020; Massicotte et al., 2021). Another study from China, including 834 patients with breast cancer, showed a prevalence of depression, anxiety and insomnia in 21.6, 15.5 and 14.7% of the cohort, respectively. A study from the US, conducted an online cross-sectional survey to compare the concerns of active cancer patients receiving treatment, to those with cancer history but not receiving treatment, as well as those with no history of cancer. Patients undergoing active treatment showed greater concern about infection from SARS-CoV2. Patients with metastatic disease felt more affected in terms of cancer care compared to patients with non-metastatic disease (50.8% vs. 31.0%, *p* = 0.02) (Lou E. et al., 2020). A study by Ahn et al. (2020), from South Korea, utilized the Six Item Stress and Anxiety to Viral Epidemics (SAVE) and Coronavirus Anxiety Scale (CAS), to screen the anxiety responses of 221 patients with breast, colorectal and gastroesophageal cancer. The study reported 49.5% patients with anxiety responses to the COVID-19 pandemic (Ahn et al., 2020). Albano et al. (2020) assessed the distress levels in lung cancer patients. Of the 441 patients, 47% showed anxiety scores of indicating a requirement for counseling. The most commonly reported reasons for distress were fear of contracting the virus, delayed testing and isolation (Albano et al., 2020). Chia et al. (2021) from Singapore, identified five themes in patients which were heightened sense of threat, impact on healthcare experience, responsibility falling on oneself, striving for normalcy and sense of safety and trust. The existence of heightened threat to COVID-19 was predominant in patients and was linked to vulnerability to COVID-19 (Chia et al., 2021). Darlington et al. (2021) from United Kingdom, recorded the experiences of parents of children with cancer. The study reported that parents no longer perceived hospital as a safe place, due to the risk of infection and expressed worry about suboptimal cancer care (Darlington et al., 2021). Another study by Younger et al. (2020) assessed the Health-related quality of life (HRQoL) and reported that 72% patients showed satisfaction over quality of care, however, the worry of COVID-19 infection was high and emotional wellbeing was affected in 41% of patients (). Lou S. et al. (2020) investigated the psychological state of HNC patients undergoing radiation therapy during COVID-19, using Symptom Checklist 90 (SCL-90), Self-Rating Depression Scale (SDS) and Self Rating Anxiety Scale (SAS). This study reported that 37.9% patients suffered severe anxiety. The total index scores decreased to 3.4% following internet based psychological intervention (Lou S. et al., 2020). A qualitative analysis by Patni et al. (2021), included 294 patients recently diagnosed with cancer or currently undergoing treatment. Of the 294, 40% had HNC and 22.4% patients indicated a delay in initiating treatment attributing it to coronavirus. In one of the surveys carried out by the Prevention and Integrative Oncology (PRIO) by the German Cancer Society, 53.8% of HNC patients showed high stress levels, expressing social isolation as the main concern (Büntzel et al., 2021).

These studies demonstrate the additive effect of COVID-19 and pre-existing cancer-related anxiety, resulting in amplification of stress in cancer patients. The subsequent sections of this review, give an overview of SARS-CoV2, the presence of its receptor ACE2 (a component of RAS) in the oral tissues, the progressive and suppressive influence of RAS on cancer (tumorigenic and anti-tumorigenic), the progressive and suppressive influence of RAS on stress (anxiogenic and anxiolytic) and finally how do stress, cancer and COVID-19 interplay through RAS, when SARS-CoV2 hijacks the ACE2 receptor.

### Overview of SARS-CoV2

The causative agent for COVID-19 is the Severe Acute Respiratory Syndrome Coronavirus 2 (SARS-CoV2) which belongs to the family Coronaviridae and order Nidovirales. Coronaviruses are large enveloped, single stranded RNA viruses that can infect humans, bats, pangolins, snakes, mice and cattle (Weiss and Navas-Martin, 2005; Wiersinga et al., 2020). It is believed that the initial mode of transmission for SARS-CoV2 was from animal to human, followed by human-to-human spread. The spread between humans occurs predominantly via respiratory droplets and contact transmission (Liu et al., 2020; Zhang J. et al., 2020). Although not a primary route of transmission, enclosed spaces with improper ventilation, aerosol generating procedures and exposure to an infected person for more than 30 min may favor airborne transmission according to the CDC guidelines (The Lancet Respiratory Medicine, 2020).

SARS-CoV2 entry into the body is mediated by the interaction of the viral spike protein with host cellular receptors. Among the host receptors, Angiotensin Converting Enzyme 2 (ACE2), is an extensively studied and recognized receptor for the entry of SARS-CoV2 (Lan et al., 2020; Sungnak et al., 2020). Later studies also revealed EMPPRIN (CD147/BASIGEN) as an important receptor and route of entry for the SARS-CoV2 virus (Radzikowska et al., 2020; Ulrich and Pillat, 2020; Wang K. et al., 2020). Spike protein on the surface of the SARS-CoV2 is cleaved at the S1/S2 site by the enzyme called Furin. Receptor binding domain (RBD) in the S1 site then binds with the receptor, ACE2, in the host cells. For SARS-CoV2 entry into a host cell, its spike protein needs to be cleaved by cellular protease, TMPRSS2 (transmembrane serine protease 2) at the S2 site, termed spike protein priming that exposes fusion peptides which fuse the cell membrane (Shang et al., 2020). The receptor-virus complex enters the cell, where it replicates, releases its contents and infects other cells (Bestle et al., 2020; Coutard et al., 2020; Hoffmann et al., 2020; Matsuyama et al., 2020; Wan et al., 2020).

Clinical manifestations have been reported to vary according to ethnicity, lifestyle factors, immune and health status (Guan et al., 2020). However, cough and fever were the initial established symptoms caused by the virus. Along with these, loss of taste and smell were later recorded using the self-reported data from the COVID-19 study app (Menni et al., 2020b). A meta-analysis by Tong et al. (2020), on the prevalence of these symptoms in COVID-19 patients reported gustatory and olfactory dysfunction as an early symptom. WHO added loss of taste and smell to the key symptoms of COVID-19 in April and United Kingdom in May (Menni et al., 2020a). Many early case studies reported the oral manifestations of COVID-19 leading to altered taste sensation, oral ulcerations, necrotizing periodontal ulcerative gingivitis, fungal infections and recurrent Herpes Simplex Virus 1 infection (Chaux-Bodard et al., 2020; Sinjari et al., 2020; Dziedzic and Wojtyczka, 2021; Martín Carreras-Presas et al., 2021; Patel and Woolley, 2021). A systematic review of ten thousand COVID-19 cases, found the presence of oral manifestations in 45% cases (Amorim dos Santos et al., 2021). Giacomelli et al. (2020), conducted a cross sectional survey of the patients admitted with SARS-CoV2 and reported taste disorders frequently appeared before serious illness and hospitalization, in most patients.

The expression of ACE2 receptors is found to be higher in Asian compared to African and American people (Batlle et al., 2020). The receptors are found in multiple organs including the heart, vessels, gut, lungs, kidney, testis and brain (Zhang H. et al., 2020). To understand the role of SARS-CoV2 receptors role in the pathogenesis of COVID-19, potential route of entry and infectivity, studies have investigated the expression of SARS-CoV2 receptors in the oral cavity (Huang N. et al., 2020). An early study in 2011, reported ACE2 positive epithelial cells lining the salivary gland ducts of rhesus macaques (Liu et al., 2011). Xu et al. (2020) analyzed bulk RNA sequence data from The Cancer Genome Atlas (TCGA) and Functional Annotation of The Mammalian Genome Cap Analysis of Gene Expression (FANTOM5 CAGE) datasets and reported a high expression of ACE2 in epithelial cells of the oral mucosa. The expression of ACE2 was found to be higher in the tongue than in buccal and gingival tissues. Lymphocytes in the oral mucosa also expressed ACE2 (Xu et al., 2020). Another study, through single cell sequence RNA database analysis, found detectable levels of ACE2 receptors in salivary glands, but lower than those in gastrointestinal tract, kidneys and heart muscles (Chen L. et al., 2020). While examining the expression of RAS components in taste buds of mice, Shigemura et al. (2019), found the presence of renin, angiotensinogen and ACE in the taste buds of circumvallate and fungiform papillae whereas, ACE2 was found in the taste buds of the papillae, as well as the tongue epithelium. Zhong et al. (2020) along with conducting single cell sequence RNA database analysis, also investigated the expression of ACE2 at the protein level, in different anatomical sites in the oral cavity by immunohistochemistry (IHC) and reported a high expression of ACE2 in the buccal mucosa, lip and tongue. This study also reported the expression of Furin in IHC samples of tongue, gingiva and lip (Zhong et al., 2020). A meta-analysis by Lechien et al. (2021) reviewed the data on expression of ACE2 and TMPRSS2 and reported expression in oral, pharyngeal and sino-nasal human mucosa. ACE2 was expressed by basal, apical, goblet, minor salivary and epithelial cells. TMPRSS2 was expressed by goblet and apical respiratory cells. The co-expression of ACE2 and TMPRSS2 was found in the olfactory region (Lechien et al., 2021).

## ACE2—The SARS-CoV2 Receptor: A Component of the Renin Angiotensin System (RAS)

**FIGURE 2:**
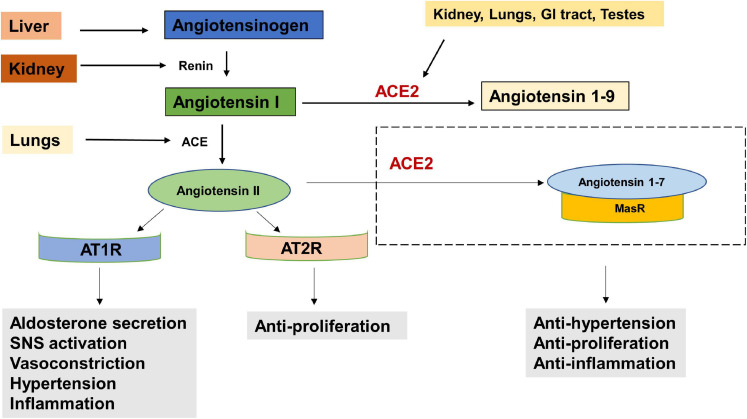
The Renin Angiotensin System (RAS). Reduced blood pressure results in the release of Renin from the kidneys which converts Angiotensinogen to Angiotensin I. Angiotensin I is physiologically inactive and a precursor to Angiotensin II. Angiotensin Converting Enzyme (ACE), produced by the lungs cleaves Angiotensin I to Angiotensin II. The binding of Angiotensin II to Angiotensin receptor Type I (AT1R) results in increased aldosterone secretion, Sympathetic Nervous System (SNS) activation, vasoconstriction, increased blood pressure and inflammation. Its binding to Angiotensin receptor Type II (AT2R) has anti-proliferative effects. Angiotensin II is converted into Angiotensin 1–7 by the enzyme Angiotensin converting enzyme 2 (ACE2) which is secreted by the kidney, lungs, GI tract, testes etc. The angiotensin 1–7/Mas1 receptor complex is considered a protective axis and has anti-hypertensive, anti-proliferative and anti-inflammatory effects.

The entry receptor of SARS-CoV2, ACE2 constitutes the protective limb of the Renin Angiotensin System (RAS). The RAS is classically well known for its physiological and pathological implications in cardiovascular homeostasis and electrolyte balance (Paz Ocaranza et al., 2020). Lately it has also been recognized for its role in the hallmarks of cancer (Wegman-Ostrosky et al., 2015). The RAS system functions through receptors- Angiotensin II Type 1 Receptor (AT1R), Angiotensin II Type 2 Receptor (AT2R), and MasR effectors- Angiotensin II, Angiotensin 1–9 and Angiotensin 1–7 and enzymes—ACE and ACE2. Expression of RAS components has been reported in liver, kidney, brain and the reproductive organs (Hunyady and Catt, 2006). Studies have also reported the presence and functionality of a local RAS in the oral cavity (Nakamura et al., 2011; Santos et al., 2015). Classically, in response to low arterial pressure or reduced sympathetic nervous system activity, the juxtaglomerular cells of the kidneys produce renin (Figure 2). Renin cleaves angiotensinogen, a hormone produced by the liver, into Angiotensin I, which is further converted to Angiotensin II, by Angiotensin Converting Enzyme (ACE). The binding of Angiotensin II to Angiotensin II Type 1 Receptor (AT1R), results in increased aldosterone production (Aguilera, 1992), sympathetic nervous system (SNS) tone (Huang et al., 2014), blood pressure (Iyer et al., 1996), vasoconstriction (Li et al., 1997), Reactive Oxygen Species (ROS) (Yamada et al., 1998) and inflammation (Wolf et al., 2002). Alternatively, the binding of Angiotensin II to Angiotensin II Type 2 Receptor (AT2R), brings about vasodilatation, reduction in blood pressure, fibrosis and inflammation (Paz Ocaranza et al., 2020). ACE2, which forms a protective axis of RAS, cleaves Angiotensin II to Angiotensin 1–7, or Angiotensin 1–9. Angiotensin 1–9 and Angiotensin 1–7 mediate their counter-regulatory and protective effects via binding to AT2R and MasR proto-oncogene, respectively (Paz Ocaranza et al., 2020).

## RAS and Cancer

### Tumorigenic Axis

**FIGURE 3:**
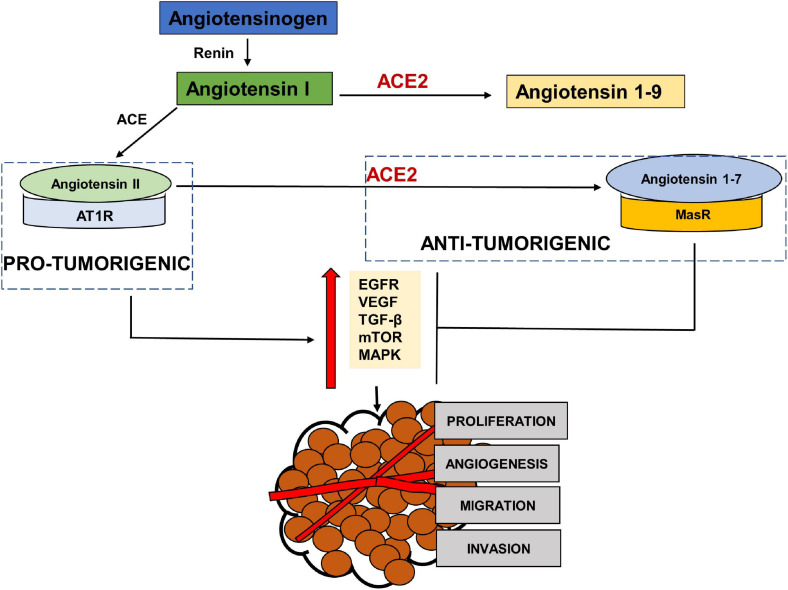
The pro-tumorigenic and anti- tumorigenic axis of the Renin Angiotensin System (RAS). Studies have shown Angiotensin II- AT1R axis signaling to activate EGFR, VEGF, TGF β, MAPK and NFKβ pathways leading to angiogenesis, migration, invasion and proliferation of tumor cells, thus acting as a pro-tumorigenic pathway. ACE2- Angiotensin 1–7- MASR acts as the anti-tumorigenic axis and blocks Ang II- AT1R induced activation of EGFR, VEGF, TGFβ, MAPK pathways.

RAS has been shown to be influential in tumorigenesis through complex interactions with several cell signaling pathways. Paracrine signaling through local RAS components may influence angiogenesis, apoptosis and proliferation (Deshayes and Nahmias, 2005; George et al., 2010; Figure 3). The main effector in tumorigenesis Angiotensin II, is produced when Angiotensin Converting Enzyme (ACE) cleaves Angiotensin I (Riordan, 2003). The binding of Angiotensin II to the Angiotensin Type 1 Receptor (AT1R) leads to an increased expression of vascular endothelial growth factor (VEGF) and epidermal growth factor receptor (EGFR) transactivation (Chua et al., 1998; Yang et al., 2005; Bhola and Grandis, 2008), increased activation of the mammalian target of rapamycin (mTOR) and mitogen-activated protein kinase (MAPK) pathways (Uemura et al., 2005; Li et al., 2016) and increased secretion of transforming growth factor beta 2 (TGFβ 2) (Daemen et al., 1991; Pallasch and Schumacher, 2020). This results in angiogenic, proliferative, anti-apoptotic and invasive tumor characteristics through the RAS. This Angiotensin II- AT1R axis therefore works as a tumorigenic axis (Escobar et al., 2004; Fujita et al., 2005; Ager et al., 2008; Figure 3).

Suppression of the pro-tumorigenic effects of Angiotensin II-AT1R by AT1R blocker has been demonstrated in many cancers, including oral cancers (George et al., 2010). Hinsley et al. (2017) reported aberrant expression of AT1R in head and neck squamous cell carcinoma (HNSCC) and the ability of Angiotensin II to promote tumor cell invasion. This study also showed that the pro-tumorigenic effects of Angiotensin II were blocked by Angiotensin 1–7 (Hinsley et al., 2017). However, binding to Angiotensin Type 2 Receptor (AT2R) shows mixed evidence with anti-tumorigenic effects in some studies (Li et al., 2009; Kawabata et al., 2012; Du et al., 2013) and pro-tumorigenic in others (De Paepe et al., 2002; Rodrigues-Ferreira and Nahmias, 2015). Matsushima-Otsuka et al. (2018) in a study on oral squamous cell carcinoma reported an increased expression of both AT1R and AT2R as the disease progressed, with a more pronounced nuclear expression of AT2R associated with tumor expression, nodal metastasis and clinical stage.

### Anti-Tumorigenic Axis

ACE2, the receptor for entry of SARS-CoV2, plays a protective role by metabolizing Angiotensin II into Angiotensin 1–7 that mediates their anti-tumorigenic effects via binding to MasR (Figure 3). These anti-tumorigenic effects of ACE2/Ang 1–7/MasR axes are through the inhibition of proliferation, angiogenesis, tumor growth and invasion (Feng et al., 2010, 2011; Xu et al., 2017). Studies in various cancers have shown that Angiotensin 1–7 induced inactivation of phosphatidylinositol 3-kinase/protein kinase B (PI3K/Akt), MAPK and VEGF signaling pathways (Machado et al., 2000; Benndorf et al., 2003; Gallagher and Tallant, 2004; Cook et al., 2010; Ni et al., 2012; Zhang et al., 2019). A profiling analysis of various cancers by Dai et al. (2020) showed a positive correlation between ACE2 expression and survival prognosis in liver cancer only. Another study using data from The Cancer Genome Atlas, found high levels of ACE2 expression in primary tumors compared to normal adjacent tissue (Winkler, 2020). A bioinformatic analysis by Huang X. et al. (2020) found an association between ACE2 and immune cell infiltration in various cancer tissues. Zhang Z. et al. (2020) also performed a computational analysis of various cancers including head and neck cancers using The Cancer Genome Atlas (TCGA) to investigate the association between expression of ACE2 receptor and oncogenic pathways, tumor phenotype and clinical outcomes. The study reported that ACE2 upregulation was associated with increased anti-tumor immune signatures and was inversely correlated with TGFβ, Wnt/β-catenin, VEGF and Notch signaling pathways. The expression of ACE2 was also inversely correlated to stemness, proliferation and epithelial mesenchymal transition (EMT). Siljee et al. (2020) investigated the expression of RAS components in cancer stem cells of head and neck malignant, metastatic melanoma and found the expression of the pro renin receptor (PRR), ACE and AT2R in all and renin in one of the tissues. ACE2 mRNA expression was reported, but none of the 20 tissues expressed ACE2 protein. Nallaiah et al. (2019) investigated the expression of RAS components in cancer stem cells of the moderately differentiated head and neck cutaneous squamous cell carcinoma. AT2R, AT1R, PRR, and ACE were expressed throughout the tumor microenvironment in all the tumor nests, peritumoral stroma and the microvessels within the tumor nests (Nallaiah et al., 2019). The expression of the RAS components was also reported in cancer stem cells of moderately differentiated squamous cell carcinoma of the lip and buccal mucosa (Featherston et al., 2016; Ram et al., 2017).

Along with ACE2, Sacconi et al. (2020) also investigated the expression of TMPRSS2 in head and neck cancer tissues using the TCGA. The study reported reduced expression of TMPRSS2 in HNC tissues. Reduced TMPRSS2 expression was associated with mutated p53 and HPV negative status of tumors (Sacconi et al., 2020). Another study using TCGA reported increased expression of ACE2 and TMPRSS2 in lung and oral cancer tissues from smokers which may indicate increased susceptibility of smokers to COVID-19 because of the increased expression of binding receptors for SARS-CoV-2, however there is not enough data from immunohistochemistry on patient tissues (Chakladar et al., 2020).

## RAS and Hallmarks of Stress

### Anxiogenic Axis

**FIGURE 4:**
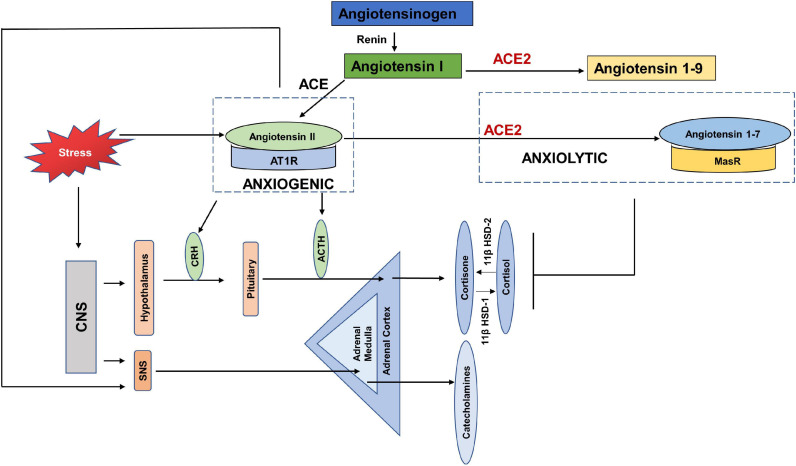
Anxiogenic and Anxiolytic axes of the Renin Angiotensin System (RAS). Increased psychological stress results in increased circulating levels of Angiotensin II, which can increase the adverse effects of Angiotensin II-/AT1R signaling. Angiotensin II/AT1R has been found to increase CRH and ACTH levels, as well as increase SNS tone and thus act as an anxiogenic axis. ACE2-/Angiotensin 1–7/MasR is found to be anxiolytic by dampening the HPA axis activation in response to stress. CNS-(Central Nervous System, CRH (Corticotropin releasing hormone), ACTH (Adrenocorticotropic hormone), SNS (Sympathetic nervous system), 11β HSD-1 (11 β hydroxysteroid dehydrogenase type 1), 11β HSD-2 (11 β hydroxysteroid dehydrogenase type 2).

Early evidence shows the link between RAS and psychogenic stress (Gaillard et al., 1981; Ganong, 1993; Figure 4). Social isolation, a consequence of both HNC and the current COVID-19 restrictions, is strongly associated with stress and is considered a risk factor for morbidity (Dickerson et al., 2011). In terms of RAS, a study also reported increased plasma renin in response to the chronic stress induced by living alone (Terock et al., 2017). Human studies investigating the response of RAS to mild acute mental stress also found increased plasma renin (Kosunen et al., 1976; Hamer et al., 2011). Gideon et al. (2020) reported that acute psychosocial stress in the form of Tier Social Stress Test (TSST), increased plasma renin, plasma and salivary aldosterone and salivary cortisol levels.

Chronic anxiety and stress are translated in the body by increased levels of pituitary and adrenal hormones, as well as catecholamines via the hypothalamus pituitary adrenal axis (HPA) and SNS (Glaser and Kiecolt-Glaser, 2005; Jacobson, 2014). Few studies have reported the direct effects of COVID-19 on the HPA axis, however, a recent study showed HPA to be rich in ACE2 receptors and TMPRSS2 (Chigr et al., 2020). Yang et al. (1996) showed that stress stimulated the circulating levels of angiotensin II. Angiotensin II has been shown to increase fear and anxiety by activating AT1R (Saavedra and Benicky, 2007; Krause et al., 2011; Marvar et al., 2014). Stress also increased the expression of AT1R and AT2R in the adrenal cortex, adrenal medulla and pituitary glands (Armando et al., 2001; Leong et al., 2002). The binding of Angiotensin II to AT1R stimulates the secretion of catecholamines, adrenocorticotropic hormone (ACTH), corticotropin releasing hormone (CRH), and corticosterone by activating the HPA axis and SNS (Ganong et al., 1987; Aguilera et al., 1995; Saavedra et al., 2005). The increased levels of glucocorticoids in response to stress, in turn increase the expression of AT1R by stimulating the GRE in the receptor promotor region (Guo et al., 1995). It was found that blockade of AT1R with AT1R antagonists, inhibited the CRH- induced ACTH and corticosterone (Armando et al., 2001; Raasch et al., 2006), and chronic mild stress (Saavedra and Benicky, 2007; Pratap et al., 2011). The Norwegian HUNT study compared the depressive symptoms of a large population of patients with systemic hypertension taking ACE inhibitors vs. those with untreated hypertension. The results showed depression-reducing effects of ACE inhibitors independent of hypertensive effects (Johansen et al., 2012). These studies show that the Angiotensin II- AT1R axis acts as anxiogenic, such that stress increases the levels of Angiotensin II and its binding with AT1R increases the levels of stress hormones (Figure 4).

### Anxiolytic Axis

Whereas studies show an anxiogenic effect of the Angiotensin- AT1R axis, ACE2 has been reported to dampen stress and anxiety related disorders via signaling through the MasR receptor and thus has an anxiolytic activity (Wang et al., 2016; Cahill et al., 2019; Figure 4). Increased levels of ACE2 were found in patients using ACE inhibitors or Angiotensin Receptor Blockers (ARB) (Furuhashi et al., 2015). Over-expression of ACE2 was linked to increased MasR receptors in the amygdala, a region of the brain that controls fear and anxiety (Davis, 1992). Wang et al. (2016), showed that over-expression of ACE2 in mice, was associated with anxiolysis by stimulating the MasR receptors. Wang et al. (2018) also showed that mice over-expressing ACE2 had lower plasma corticosterone levels, blunted HPA axis activation and reduced anxiety-like behavior. Studies have shown that ACE2/Angiotensin 1–7/MasR represents a promising therapeutic target for treatment of anxiety disorders and depression.

## How Do Stress, Cancer, and RAS Interplay When SARS-CoV2 Hijacks ACE2?

The presence of SARS-CoV2 in the community and merely the thought of being infected, may generate fear and anxiety, the full impact of which may only unfold with the passage of time. The studies referenced above suggest that the COVID-19 pandemic has increased stress levels in the general population, as well as in patients with cancer. However, there might appear to be a dearth of studies aimed specifically at patients with HNC, to understand the effects of stress on disease progression during the COVID-19 pandemic, and any resulting effect on symptoms.

Many studies have shown increased levels of circulating catecholamines and cortisol in HNC (reviewed in Table 1, Iftikhar et al., 2021). In terms of COVID-19, levels of cortisol and HADS score were significantly increased in patients who died of COVID-19 (Ramezani et al., 2020). Studies have also pointed out the possibility of using cortisol levels as biomarkers of serious illness due to COVID-19 (Tan et al., 2020).

**FIGURE 5:**
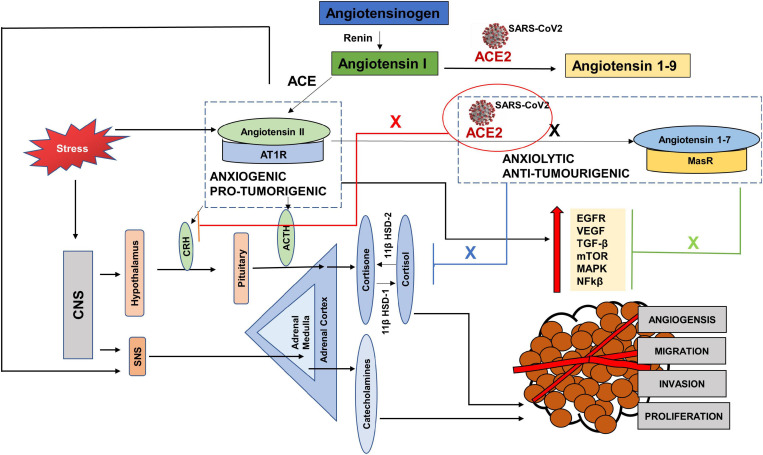
The interplay between SARS CoV-2, cancer progression and stress via the Renin Angiotensin System (RAS). Hijacking of ACE2 receptor by SARS-CoV2 may compromise the protective arm of the Renin Angiotensin System (RAS), by creating a deficiency of ACE2. As a result, there is an increased level of Angiotensin II, since it is not cleaved by ACE2 to Angiotensin 1–7 (black cross). This causes an increase in the pro-tumorigenic and anxiogenic- Angiotensin II- AT1R signaling, which increases the stress hormones and activates the oncogenic pathways. These pathways not only progress the tumor but are also used for replication of SARS-CoV2. Again, due to the deficient ACE2, the axis- ACE2/Angiotensin 1–7/MasR cannot counteract the effects of ANGII-AT1R signaling and unable to block the oncogenic pathways (Green cross) as well as the increased levels of stress hormones (blue cross) and (red cross).

SARS-CoV2 uses both the protective and adverse arms of the RAS to its advantage. To gain access into the body, it must bind to the ACE2 receptor. Once it is inside, ACE2 is internalized and becomes downregulated to carry out its protective anti-tumorigenic and anxiolytic functions and as a result there are increased levels of Angiotensin II with increased AngII-AT1R signaling (Figure 5). Since the evidence of ACE2 expression in tumors is mixed, with some studies showing increased expression, while others showing decreased expression, it is easy to speculate that low expression may act as a COVID-19 protective mechanism for cancer patients (Siljee et al., 2020; Winkler, 2020). However, at the same time, conditions with deficient ACE2 already, may pose a greater threat if the available ACE2 also gets hijacked.

To replicate, SARS-CoV2 requires EGFR, PI3K, MAPK, TGFβ and nuclear factor kappa-light-chain-enhancer of activated B cells (NFkβ) pathways (Hemmat et al., 2021), activated by the adverse arm, Angiotensin II- AT1R, which has anxiogenic and pro-tumorigenic effects (Chua et al., 1998; Wolf et al., 2002; Hunyady and Catt, 2006; Bhola and Grandis, 2008). Increased circulating levels of Angiotensin II increase the levels of cortisol and SNS activation. These stress hormones further aid tumor progression and replication of the virus directly, by activating some of the pathways and by increasing the levels of Angiotensin II, leading to the angiotensin II-AT1R signaling (Figure 5).

**FIGURE 6:**
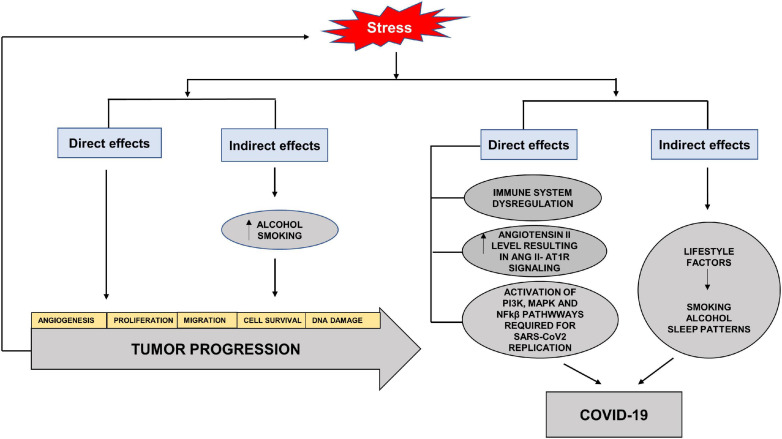
Direct and indirect effects of stress on tumor progression and COVID-19. Stress hormones have direct effects on cancer progression by increasing angiogenesis, cell proliferation, migration, cell survival and DNA damage. The indirect effects of stress may be seen in the adoption of habits such as increased alcohol consumption and cigarette smoking, which aid cancer progression. The direct effects of stress on COVID-19 are through immune dysregulation, increased Angiotensin II levels and activation of pathways required for SARS-CoV2 replication. The indirect effects involve adoption of unhealthy lifestyle factors such as increased alcohol intake, smoking and altered sleep patterns.

Stress has always been an unavoidable element in the balance between health and disease. The SARS-CoV2 virus reminds us of the direct and indirect effects of stress on different systems and diseases. Studies have highlighted the role of stress hormones in cancer progression by influencing cell proliferation, angiogenesis, invasion, migration, cell survival, immune dysregulation and DNA damage (Iftikhar et al., 2021). The indirect effects of stress may be seen in the adoption of habits such as increased alcohol consumption and cigarette smoking, which aid cancer progression (Figure 6). Studies have shown that cortisol released by stress-induced HPA axis dysregulation is associated with alcohol misuse (Dunlavey, 2018). Although HNC are considered to be amongst the most stressful cancers and closely associated with alcohol and smoking, surprisingly studies do not highlight stress as a direct or indirect factor in the etiology or progression of HNC.

In terms of COVID-19, stress serves to elevate circulating levels of Angiotensin II and aids in the activation of pathways needed for replication and increase in the viral load (Yang et al., 1996). The effects of stressors on the immune system and diminished response to vaccines have also been seen in viral infections (Glaser and Kiecolt-Glaser, 2005). Acute stress has been shown to activate NFkβ (Kuebler et al., 2015). Studies have also shown the stress hormones to activate PI3K and MAPK via cyclic adenosine monophosphate (cAMP), activated protein kinase A (PKA) and exchange factor directly activated by cAMP (EPAC) effector pathways, reviewed in Iftikhar et al. (2021). The indirect effects can lead to the adoption of unhealthy lifestyle with altered sleeping patterns, alcohol misuse and smoking which increase the susceptibility to infection, as well as decrease the effectiveness of vaccines (Glaser and Kiecolt-Glaser, 2005; Figure 6).

## Conclusion

Deteriorating mental health was already a challenge of the twenty-first century and has now been amplified by the pandemic that has changed the world its people were accustomed to. The adaptation to the new normal has been an additional source of emotional turmoil for the general population but more so for cancer patients who not only had to reset their way of living, but also deal with the disruption in their diagnosis and ongoing treatment. In the last two decades, scientific activity has increased the efforts to determine the effect of stress on cancer progression, but comparatively fewer studies are seen in HNC even though they are considered to be amongst the most stressful cancers due to the anatomical, cosmetic and functional importance of this region. With the pandemic hitting the world and affecting mental health, the need to understand the impact of psychological stress on cancer progression has been underscored.

Through this review we found that fear and anxiety has been/is being widely explored in cancer patients during the pandemic, yet again only a few studies have reported the impact of the pandemic on the mental health of patients with HNC so far. In addition to the direct effects that stress may have on COVID-19 and cancer, it also reminds us of the indirect effects that lead to lifestyle choices which progress COVID-19 and HNC.

In the light of the referenced studies, this review summarized the interaction between RAS and cancer, RAS and stress and presents an insight on how stress, cancer and COVID-19 may interact mechanistically through RAS and facilitate each other’s progression when SARS-CoV2 hijacks ACE2. As a result of this hijacking, the protumorigenic and anxiogenic signaling through Angiotensin II- AT1R is increased, which in turn may lead to the activation of signaling pathways required for SARS-CoV2 replication.

Considering HNC are psychologically very stressful, there is an urgent need to investigate the impact of COVID-19 on stress burden in patients with HNC and expansion of strategies for its management. Furthermore, studies may look at how this increased psychological stress has impacted disease progression and which symptoms have been affected the most. Alongside, *in vitro* studies are also important to determine if increased levels of stress affect the expression of the entry receptor for SARS-CoV2 in HNC tissues and cells, to establish if stress makes HNC more prone to COVID-19 in addition to its effect on the immune system and lifestyle.

## Author Contributions

AI, MI, SS, SJ, and IE: conceptualization and writing—review and editing. AI: methodology. AI and MI: software and investigation. AI, SJ, and IE: writing—original draft preparation and project administration. IE, SJ, and SS: supervision. SJ and IE: funding acquisition. All authors have read and agreed to the published version of the manuscript.

## Conflict of Interest

The authors declare that the research was conducted in the absence of any commercial or financial relationships that could be construed as a potential conflict of interest.

## Abbreviations

ACE: Angiotensin converting enzyme
ACE2: Angiotensin converting enzyme 2
ACTH: adrenocorticotropic hormone
ARB: angiotensin receptor blockers
AT1R: angiotensin II type 1 receptor
AT2R: angiotensin II type 2 receptor
cAMP: cyclic adenosine monophosphate
CAS: coronavirus anxiety scale
COVID-19: coronavirus disease 2019
CRH: corticotropin releasing hormone
EGFR: epithelial growth factor receptor
EMT: epithelial mesenchymal transition
EPAC: exchange factor directly activated by cAMP
GAD-7: generalized anxiety disorder questionnaire
HAD-A: hospital anxiety and depression scale
HADS-D: hospital anxiety and depression scale
HNC: head and neck cancer
HRQoL: health related quality of life
HPA: hypothalamus pituitary adrenal axis
IES-R: Impact of events scale revised
MAPK: mitogen activated protein kinase
NFk β: nuclear factor kappa-light-chain-enhancer of activated B cells
PI3K/AKT: phosphatidylinositol 3-kinase/protein kinase B
PKA: protein kinase A
PTSD: post-traumatic stress disorder
RAS: renin angiotensin system
RBD: receptor binding domain
ROS: reactive oxygen species
SARS-CoV2: severe acute respiratory syndrome corona virus
SAS: self- rating anxiety scale
SAVE: stress and anxiety to viral epidemics
SCL-90: symptom checklist 90
SDS: self-rating depression scale
SNS: sympathetic nervous system
TMPRSS2: transmembrane protease serine 2
TCGA: the cancer genome atlas
TGF β 2: transforming growth factor beta 2
VEGF: vascular endothelial growth factor.
